# Pyoderma gangrenosum after caesarean section: a case report

**DOI:** 10.1186/1742-4755-3-9

**Published:** 2006-08-22

**Authors:** Franyke Banga, Nico Schuitemaker, Piet Meijer

**Affiliations:** 1 Obstetrics and Gynaecology, Vrije Universiteit Medical Center, Postbox 7057, 1007 MB, Amsterdam, The Netherlands; 2 Obstetrics and Gynaecology, Diakonessenhuis, Utrecht, The Netherlands; 3 Dermatology, Diakonessenhuis, Utrecht, The Netherlands

## Abstract

**Background:**

Pyoderma gangrenosum is a rare ulcerative skin disease. The diagnosis is based on clinical features and excluding other causes of skin ulcers, as it does not have characteristic histopathology or laboratory findings. The etiology is poorly understood. Lesions can develop spontaneously, after surgery or after trauma.

**Case presentation:**

We present the case of a 32-year-old woman with ulcerative wound defect after caesarean section. The wound was not healing despite standard wound care and antibiotic treatment. Pyoderma gangrenosum was diagnosed and after high dose corticosteroids wound healing started.

**Conclusion:**

Early diagnosis and subsequent treatment of pyoderma gangrenosum are crucial for limiting scar tissue. Diagnosis of pyoderma gangrenosum could easily be missed since gynaecologists are rarely confronted with this disorder.

## Background

Pyoderma gangrenosum is a rare skin disease. Classical clinical presentation is a first development of an erythematous papule or pustule, secondary forming a rapidly progressing, painful cutaneous ulcer with characteristic violaceous colored borders. Lesions can develop spontaneously, after surgery or after minor trauma [1]. Pyoderma gangrenosum was first described in 1930 by Brunsting et al [2]. The etiology is poorly understood but immune system abnormalities have been reported. Diagnosis is based on clinical features and excluding other causes of skin ulceration [1,3]. Obstetrical or gynaecological reports about pyoderma gangrenosum are rare. We present a woman with pyoderma gangrenosum after caesarean section.

## Case presentation

A 32 year old woman was regularly seen at our antenatal outpatient clinic during her first pregnancy (gravida 1 para 0). Her medical history revealed cervical intraepithelial neoplasia (CIN II), which was treated 2 years earlier by loop excision and a bilateral breast reduction with complicated but spontaneous wound healing 13 years earlier.

At 18 weeks of gestation, the woman was diagnosed with chronic hypertension, with maximum diastolic blood pressure of 95 mm Hg. At 31 weeks (+ 4 days) she presented with lower abdominal pain and fever. Two weeks before she had experienced a period of flu like symptoms, headache and a sore throat. Physical examination showed a body temperature of 38.3°C, uterine contractions and a fundal height corresponding to 29 weeks of gestation. The cervix was 1 cm dilated and completely effaced; the amniotic membranes were intact. Ultrasound indicated a fetal growth corresponding to the fifth percentile and oligohydramnios. Fetal cardiotocogram (CTG) showed a variable fetal heart rate of 160 beats per minute and late decelerations. External tocodynamometry registrated uterine contractions. Blood tests revealed a high white blood cell count (WBC) of 24,4 × 10.9/L with neutrophilia and an elevated C-reactive protein (CRP) of 170 mg/L. After performing vaginal culture, intravenous amoxicillin clavulanate and ritodrine were started. The contractions diminished, but fetal heart rate decelerations were still present. Caesarean section for suspected fetal distress was performed and a healthy boy of 1227 gram (5^th^–10^th ^percentile) was born with 1 and 5-minute Apgar scores of 9 and 10, respectively.

**Figure 1:**
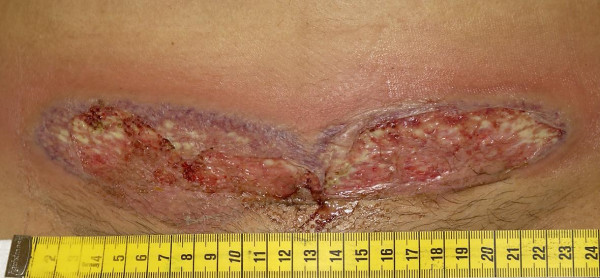
Twenty-three days after delivery.

Postoperatively, the woman continued to receive amoxicillin clavunalate – her temperature normalised and CRP dropped to 84 mg/L. On day 5 postpartum a spiking fever developed. Physical examination of the lungs, breasts and wound were not suspicious for infection. CRP rose to 321 mg/L and WBC was 27.5 × 10.9/L. Cultures taken from the vagina and throat, and urine and stool cultures were negative. Serologic tests for toxoplasmosis, syphilis, rubella, cytomegalovirus and herpes simplex virus were negative. On day 8 postpartum the woman had still a spiking fever and a raised CRP despite amoxicillin clavunalate. Abdominal ultrasound and X-ray of the thorax were normal. Amoxicillin clavunalate was discontinued. Nine days after delivery, the Pfannenstiel wound developed purulent discharge and a small peri-incisional erythema; the wound borders were closed. On day 11 postpartum a wound culture was indicative for staphylococcus aureus and flucloxacillin was started. Definite culture was negative and flucloxacillin was changed to oral clindamycin on day 17 postpartum. The wound was now more erythematous, had blue-red colored margins, was infiltrated and gaping at the corners. CRP was 85 mg/L and WBC 17.7 × 10.9/L. Standard conservative wound care was advised and the woman was discharged to home care 19 days after delivery. Twenty-three days after caesarean section, the woman was readmitted because of severe wound pain. CRP was 409 mg/l and WBC 34.1 × 10.9/L. Violaceous coloured wound borders were spread expanding the ulcerative defect progressively (Figure 1). Histopathologic findings of the wound biopsy showed necrosis of the epidermis with edema and bulla and an intense infiltration of granulocytes in the dermis. There were no signs of vasculitis and immunofluorescence assay was negative for immune complex vasculitis.

The clinical features and histopathological findings made pyoderma gangrenosum highly probable. Prednisone 65 mg/day (1 mg/kg) was initiated. Paracetamol, nonsteroidal anti-inflammatory drugs and tramadol were prescribed for pain relief. Because the wound defect did not stabilise, prednisone was increased to 100 mg/day orally. Wound borders were treated with tacrolimus 0.1 % cream. Standard wound care consisted of gel and cutarazine gaze. Within 48 hours the wound defect stabilised and CRP decreased. Wound culture was positive for pseudomonas aeruginosa and was treated with topical acetic acid 1%.

**Figure 2:**
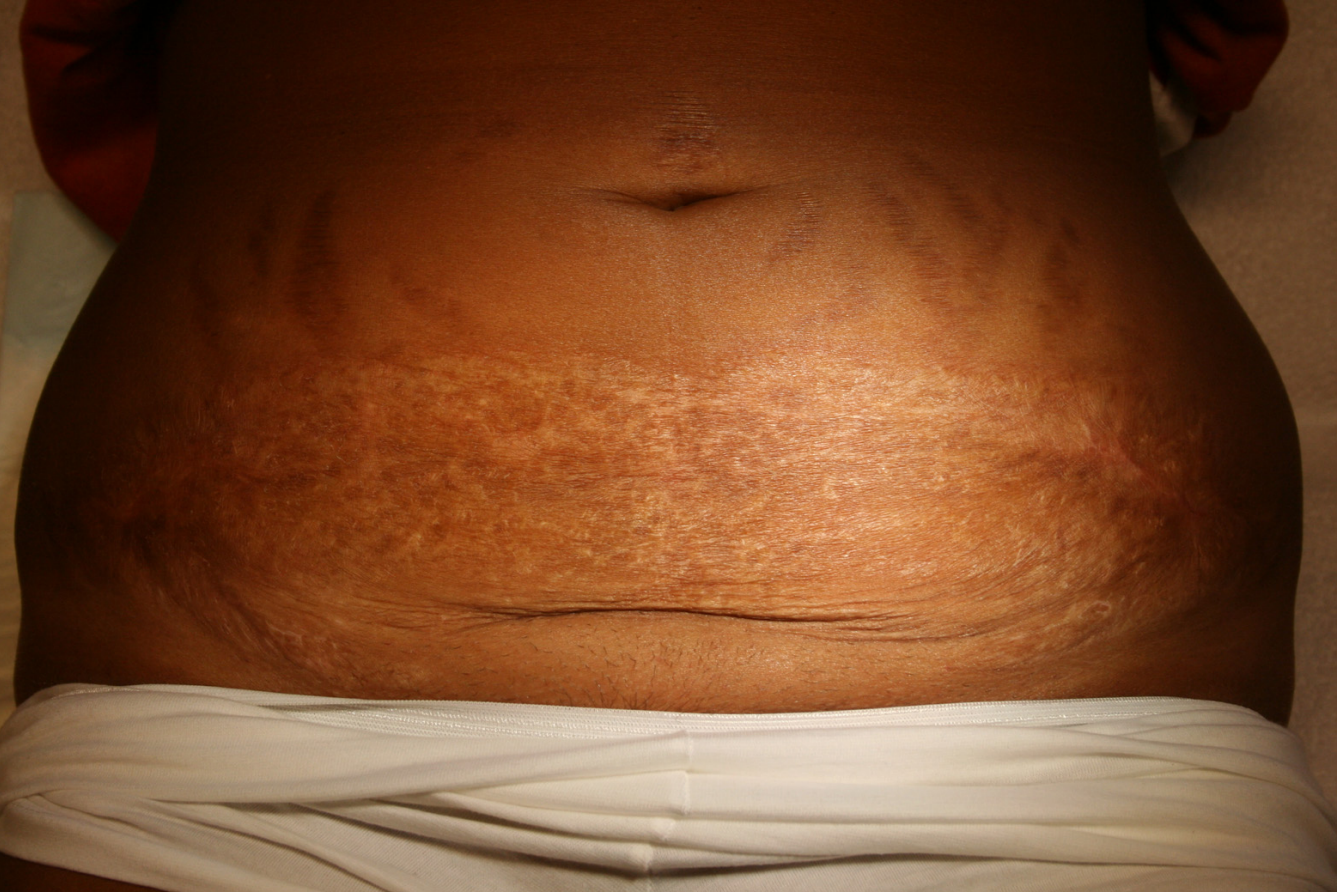
Six months after delivery.

Twenty-three days after readmission and 46 days after caesarean section, the woman was discharged on prednisone 80 mg/day. Two months after delivery wound healing was incomplete and prednisone 40 mg was maintained till 6 months after delivery. At this time the wound was completely healed leaving a large "parchment paper" scar (Figure 2). Prednisone was decreased by 5 mg a week and could be stopped almost 9 months after caesarean delivery.

Serum protein electrophoresis was normal, antiphospholipid, antinuclear and antineutrophil cytoplasmic antibodies and rheuma factor were negative. The woman's family history showed familial polyposis but colonoscopy indicated no abnormality.

## Discussion

Pyoderma gangrenosum is a rare ulcerative skin disease. It is classified under the neutrophilic dermatoses. Lesions can develop spontaneously, after surgery or after minor trauma. The latter form is known as the pathergy phenomenon. Lesions can be single or multiple, chronic or recurrent. There can be severe wound pain, fever and malaise. The classical presentation is the development of an erythematous papule or pustule that breaks down to form an ulcer with purulent discharge, and violaceous colored borders spreading peripherally and overhanging the ulcer bed. Especially early stages resemble a bacterial infection. Classical signs are the ineffective standard wound care and antibiotic treatment. Surgical debridement can make the defect even worse (pathergy) [1]. No laboratory finding is diagnostic of pyoderma gangrenosum, but neutrophilic leukocytosis and elevated erythrocyte sedimentation rate are often found. Early histopathological feature is dermal neutrophilic abscess; later biopsies demonstrate epidermal necrosis and ulceration, superficial dermal edema and a dense, mixed dermal infiltrate [3]. Histopathologic findings are not specific but crucial to rule out other causes of skin ulcers. The differential diagnosis of skin ulcerations consists of primary infection (bacterial, fungal and viral), vasculitis, malignancy, vascular occlusive or venous disease, drug-induced or exogenous tissue injury and other inflammatory disorders [1,3]. These pyoderma-gangrenosum-resembling ulcers can lead to misdiagnosis which is not uncommon. Weenig et al. [3] reviewed 240 patients with a diagnosis of pyoderma gangrenosum and reported approximately 10 % of misdiagnosis, exposing patients to risks associated with its treatment. All patients with suspected pyoderma gangrenosum should have an intensive follow-up with re-evaluation of the diagnosis including consideration of repeat skin biopsy if no response to treatment [3].

The pathophysiology of pyoderma gangrenosum is unknown, but altered immune system is believed to be involved. Most common is an association with systemic diseases especially arthritides (seronegative and seropositive), inflammatory bowel disease and haematological disorders (leukemia and monoclonal gammapathies). Less common associated diseases include hepatic diseases, myelomas, immunological diseases and HIV infection. Screening tests for underlying diseases should be performed [1].

Based on empirical findings, first choice of treatment is systemic high dose corticosteroids. A dramatic improvement with corticosteroid treatment supports the diagnosis of pyoderma gangrenosum. In mild cases topical therapy might be sufficient. First choice of alternative systemic treatment, for example in corticosteroid resistant cases or severe corticosteroid side effects, is cyclosporine A [4]. Other alternative therapeutic procedures, reported in case reports, are mycophenolate mofetil, tacrolimus, infliximab or plasmapheresis [4].

Pyoderma gangrenosum after abdominal surgery is most commonly reported in patients with inflammatory bowel disease following peristomal colostomy [1]. Pyoderma gangrenosum has also been described several times after breast reduction [5].

Obstetrical or gynaecological reports about pyoderma gangrenosum are rare.

Only four cases of pyoderma gangrenosum after caesarean delivery have been reported before [6-9]. Stone et al. described a previously healthy 36-year-old primigravida developing pyoderma gangrenosum after emergency caesarean section for fetal distress at 29 weeks of pregnancy [6]. Steadman et al. reported pyoderma gangrenosum after caesarean section for breech presentation in a 32-year-old woman. After the pyoderma gangrenosum developed, immunoglobulin electrophoresis revealed hypogammaglobulinaemia that resolved after steroid treatment [7].

Ronnau et al. described a 24-year-old woman with a history of wound healing defects after appendectomy and cicatricotomy treated with immune globulin. A caesarean section due to cephalopelvic disproportion was complicated by pyoderma gangrenosum diagnosed 29 days postpartum and treated with high dose prednisolone. A chronic hepatitis C virus infection was revealed [8]. All patients mentioned above were non-responsive to antibiotic treatment and rapidly improved after high dose of corticosteroids.

There are two other gynaecological cases of pyoderma gangrenosum after laparotomy with transverse incision: Keohane et al. [10] described a 46-year-old woman undergoing hysterectomy and salpingo-oophorectomy because of uterine fibroids; Budak et al. [11] reported a 27-year-old woman having a cystectomy for endometrioma. Both cases were negative for underlying diseases. We found two case reports about cervical pyoderma gangrenosum; both patients were without associated diseases [12,13]. Vulvar pyoderma gangrenosum has been reported six times, most of them had underlying diseases such as inflammatory bowel disease or haematologic disorders [14].

## Conclusion

When a postoperative ulcerative wound defect is not healing despite standard wound care, antibiotic treatment and negative cultures, pyoderma gangrenosum should be suspected. Early diagnosis and subsequent treatment are crucial for limiting scar tissue. Diagnosis of pyoderma gangrenosum could easily be missed since gynaecologists are rarely confronted with this disorder.

## Competing interests

The author(s) declare that they have no competing interests.

## Authors' contributions

FB (resident gynaecology), NW (gynaecologist) and PM (dermatologist) were consulted for diagnosis and treatment of the patient. All authors participated in the preparation of the manuscript, read and approved the final manuscript.
